# 超声提取-气相色谱-质谱法识别和测定焚香释放气体及颗粒物中的有机化合物

**DOI:** 10.3724/SP.J.1123.2024.10022

**Published:** 2025-07-08

**Authors:** Ziqi YUE, Lu JIANG, Zhigang LI, Wei WANG, Yawei WANG

**Affiliations:** 1.中国科学院生态环境研究中心，环境化学与生态毒理学国家重点实验室，北京 100085; 1. State Key Laboratory of Environmental Chemistry and Ecotoxicology，Research Center for Eco-Environmental Sciences，Chinese Academy of Sciences，Beijing 100085，China; 2.国科大杭州高等研究院环境学院，浙江 杭州 310024; 2. School of Environment，Hangzhou Institute for Advanced Study，University of Chinese Academy of Sciences，Hangzhou 310024，China; 3.中国科学院大学，北京 100049; 3. University of Chinese Academy of Sciences，Beijing 100049，China

**Keywords:** 气相色谱-质谱, 焚香产物鉴定, 有机化合物, 室内空气, gas chromatography-mass spectrometry （GC-MS）, incense products identification, organic compounds, indoor air

## Abstract

焚香会造成室内空气污染，长期暴露于焚香环境中会对人体健康构成风险。本研究基于超声提取-气相色谱-质谱（GC-MS）法，建立了可同时测定焚香释放的气体和颗粒物中67种有机化合物（包括29种酯类化合物、7种苯系物、14种酚类化合物和17种多环芳烃）的分析方法。研究利用石英滤膜和自制的XAD-2树脂采样管分别收集室内焚香释放的颗粒态和气态组分，并将收集到的两类样品分别进行超声提取。利用气相色谱-四极杆-静电场轨道阱组合式高分辨质谱（GC-Q-Orbitrap-HRMS）进行焚香成分的非靶向分析，以此实现对焚香样品中痕量有机化合物的初步鉴定；之后利用GC-MS在选择离子监测（SIM）模式下进行靶向分析，用外标法定量。方法学验证结果表明，67种有机化合物在10~500 μg/L范围内线性关系良好，相关系数（*r*）均≥0.999 0，检出限（LOD）为0.02~0.33 µg/m^3^，定量限（LOQ）为0.03~0.67 µg/m^3^。在低、中、高3个加标水平下，67种有机化合物在气态组分中的回收率为72.7%~119.0%，相对标准偏差（RSD）为0.9%~4.1%；67种有机化合物在颗粒态组分中的回收率为71.5%~118.9%，RSD为0.7%~9.5%。将所建方法应用于室内焚香样品（线香和电加热香粉）的测定，并分析了不同焚烧方式对产物种类及含量的影响。结果表明，在相同的焚香时间（30 min）内，线香点燃时释放的气态和颗粒态有机化合物总含量均高于电加热香粉。线香燃烧产生的颗粒态有机化合物以酚类化合物和多环芳烃为主，其次是酯类化合物，苯系物仅占很少一部分；电加热香粉产生的颗粒态有机化合物组成与线香相似。在焚烧线香释放的气态有机化合物中检测到了大量的苯系物，含量远超其他3类有机化合物；电加热香粉释放的气态有机化合物以苯系物和酯类化合物为主，其次是多环芳烃和酚类化合物。与先前报道的相关监测方法相比，本方法更为简单、高效，为焚香释放的气态和颗粒态有机化合物的灵敏筛检提供了更便捷的技术手段。

## Abstract (en)

Statistical evidence indicates that individuals spend approximately 90% of their time indoors on a daily basis. A multitude of human activities， including incense burning， cooking， smoking， and the use of electrical appliances， can contribute to indoor air pollution. This phenomenon poses a significant risk of indoor exposure to human health risks. Incense burning， as one of the major indoor pollution sources， has been increasing in use in recent years due to its special effects in religious practices and regional lifestyles. Incense usually consists of herbs， wood powder， bamboo sticks， scented materials， and binder powders， and the specific chemical composition of each type of material is complex due to different uses and manufacturing processes； and the degree of complete combustion， thermal degradation and volatilization processes of different types of incense vary greatly， resulting in the release of gaseous and particulate organic compounds whose compositions are not yet clear. Studies have shown that the emission factors of particulate matter released from incense burning may be much higher than those produced during the combustion of charcoal， wood and cigarettes. The levels of organic pollutants emitted by incense burning， such as aromatic hydrocarbons， esters， ketones， benzenes， and phenols， are much higher than outdoor levels. Prolonged exposure to incense smoke has been shown to cause respiratory damage and potential genotoxicity. Therefore， accurate identification and measurement of the organic pollutants emitted from incense burning is particularly important to accurately assess their environmental and population health risks. Herein， this study established an analytical method based on ultrasonic extraction-gas chromatography-mass spectrometry （GC-MS） for the simultaneous determination of 67 organic compounds in gaseous and particulate emissions from incense burning. These compounds include 29 esters， seven benzene series， 14 phenols， and 17 polycyclic aromatic hydrocarbons （PAHs）. Particulate and gaseous components released during indoor incense burning were collected using quartz filter membrane and self-made XAD-2 resin sampling tubes， respectively. Non-targeted analysis of incense components was performed using a GC-quadrupole-Orbitrap high-resolution mass spectrometry （GC-Q-Orbitrap-HRMS） to achieve preliminary identification of trace organic compounds in the incense-burning samples. Subsequently， targeted analysis was conducted using GC-MS in selected ion monitoring （SIM） mode， with quantification performed using the external standard method. Method validation results demonstrated good linearity for all 67 organic compounds within the range of 10 to 500 μg/L， with correlation coefficients （*r*）≥0.999 0. The limits of detection （LODs） and quantification （LOQs） were ranged from 0.02 to 0.33 µg/m³ and 0.03 to 0.67 µg/m³， respectively. At low， medium， and high spiked levels， the recoveries for the 67 organic compounds in the gaseous phase ranged from 72.7% to 119.0%， with relative standard deviations （RSDs） of 0.9% to 4.1%； for the particulate phase， the recoveries for the 67 organic compounds ranged from 71.5% to 118.9%， with RSDs of 0.7% to 9.5%. The established method was applied to the determination of indoor incense-burning samples （incense sticks and electrically heated incense powder）， and the influence of different incineration methods on the types and contents of products was analyzed. The results indicated that during the same burning duration （30 min）， the total content of gaseous and particulate organic compounds released from ignited incense sticks was higher than that from electrically heated powdered incense. The particulate organic compounds produced by the ignited incense sticks are mainly phenolics and PAHs， followed by esters， and benzene series. The composition of particulate organic compounds from electrically heated powdered incense was similar to that of incense sticks. A large amount of benzene series were detected in the gaseous organic compounds released by burning incense sticks， and the content was much higher than that of the other three types of organic compounds. The gaseous organic compounds released by electrically heated incense powder are mainly benzene series and phenols， followed by PAHs and esters. Compared with the previously reported related monitoring methods， this method is simpler and more efficient， providing a more convenient technical means for the sensitive screening of gaseous and particulate organic compounds released by incense burning.

据统计，在现代社会人们平均每天约有90%的时间处于室内环境^［1］^。焚香、烹饪^［2］^、吸烟以及电器使用等一系列人为活动均可能成为室内空气污染源，进而对人体健康造成严重的室内暴露风险^［3，4］^。焚香作为室内主要污染源之一，其在宗教活动以及地域生活方式中具有特殊意义和功效^［5］^，近年来其使用量和使用频率均呈日益增长态势^［6］^。香通常由草药、木粉、竹棒、香味材料和黏合剂粉等成分构成。由于不同香在用途和制造工艺上存在差异，每类材料中所包含的具体化学成分较为复杂^［7］^；同时，不同种类香的燃烧完全程度、热降解过程以及挥发特性也存在较大差别^［8］^，这使得焚香过程中释放的气态和颗粒态有机化合物的具体组成尚未完全明晰^［9］^。研究表明，焚香过程中释放的颗粒物排放因子可能远高于木炭、木材和香烟等材料燃烧时的排放因子^［10］^。焚香排放的有机污染物种类繁多，包括芳烃类^［11，12］^、酯类^［13］^、酮类^［14］^、苯系物、酚类^［15］^等，这些污染物的含量远高于室外空气中的含量值^［16］^。Yadav等^［17］^研究发现，寺庙内空气中多环芳烃（PAHs）的含量约为室外空气的27倍。已有研究证实，长时间接触焚香烟雾会对呼吸系统造成损伤，并可能产生潜在的遗传毒性^［17，18］^。因此，精准识别和测定焚香烟雾中有机污染物的排放源谱，对于准确评估其带来的环境及人群健康风险至关重要。

在焚香过程中产生的颗粒态有机化合物，通常采用滤膜进行捕集，常用的滤膜类型包括石英滤膜、聚氨酯软性泡沫（PUF）、玻璃纤维滤膜（GFF）以及聚四氟乙烯膜（PTFE）等。其中，石英滤膜凭借其对颗粒态有机化合物捕集效率高、本底值低且耐高温（>900 ℃）等优势，已成为目前最常用的收集材料^［19，20］^。对于气态化合物，通常依据挥发性有机化合物的含量和性质，采用固体吸附剂（如Tenax GC、Tenax TA、XAD-2树脂等）进行采集，或使用容器捕集法（如气袋、苏玛罐）进行收集^［21，22］^。焚香过程中产生的气态和颗粒态有机化合物可采用超声提取、加速溶剂萃取或索氏提取等方法进行提取^［23］^。加速溶剂萃取对设备和耗材的要求较为严苛，索氏提取的耗时较长，而超声提取因具备快速、成本低廉且高效的特性，具有更为广泛的应用范围。由于室内环境空气中有机化合物组成复杂，针对每一类化合物都有其合适的测量仪器。例如，依据《环境空气 酚类化合物的测定 高效液相色谱法》（HJ 638-2012）^［24］^，酚类物质的测定需使用高效液相色谱仪；按照《电子烟》（GB 41700-2022）^［25］^的规定，酯类物质的测定同样采用高效液相色谱仪；而《环境空气 苯系物的测定 固体吸附/热脱附-气相色谱法》（HJ 583-2010）^［26］^则要求使用配有氢焰离子化检测器（FID）^［27］^的气相色谱仪来测定苯系物。随着质谱技术的不断发展，针对单独一类污染物进行精准定量的优化程度日益提升。然而，目前仍缺乏简单快速识别环境空气中多种类有机化合物的方法。气相色谱-质谱（GC-MS）是大多数实验室都配备的基础气相分析仪器，因此可基于该仪器建立靶向分析方法。近年来，随着高分辨质谱技术的不断发展，一些非靶向分析方法，如气相色谱-四极杆-飞行时间质谱（GC-Q-TOF MS）、全二维气相色谱-飞行时间质谱（GC×GC-TOF MS）、气相色谱-四极杆-静电场轨道阱组合式高分辨质谱（GC-Q-Orbitrap-HRMS）^［28，29］^，正越来越多地应用于有机化合物的检测中。GC-Q-Orbitrap-HRMS具有高分辨率和高灵敏度的特点，能够提供碎片离子元素组成的精确质量百分比以及多种电离模式下的质谱碎片信息，其抗干扰能力强，可对复杂样品基质中的痕量化合物进行准确检测^［30‒32］^。

本研究基于Orbitrap Exploris GC建立了针对焚香释放气体及颗粒物中有机化合物的非靶向分析方法。在此基础上，利用超声提取-GC-MS法，建立了可同时测定焚香释放的67种有机化合物（包括29种酯类化合物、7种苯系物、14种酚类化合物和17种多环芳烃）的靶向分析方法。该方法具备便捷、准确、灵敏等特点，将其应用于实际藏香样品的定性与定量检测，取得了良好效果，为焚香产物中颗粒态及气态有机化合物的灵敏筛检提供了新的方法和技术。

## 1 实验部分

### 1.1 仪器、试剂与材料

GCMS-QP2020NX气相色谱-质谱联用仪、TQ 8050 NX气相色谱-三重四极杆质谱联用仪（日本岛津公司）；Orbitrap Exploris GC 240气相色谱-四极杆-静电场轨道阱组合式高分辨质谱联用仪（美国Thermo公司）；KQ-500VDE双拼数控超声波清洗器（昆山市超声仪器有限公司）；N-EVAPTM111氮吹仪（美国Organomation Associates公司）；LT 24/12马弗炉（德国Nabertherm公司）；47 mm石英滤膜、0.45 µm 聚四氟乙烯（PTFE）滤膜（美国Pall公司）；SU853005-Amberlite XAD-2大孔吸附树脂（德国Merck公司）；流量可调采样器（常德比克曼生物科技有限公司）。

67种有机化合物标准品（纯度均≥94.4%）均购自北京曼哈格生物科技有限公司。正己烷（色谱纯，德国Merck公司）；二氯甲烷（色谱纯，美国ACS恩科化学公司）；丙酮（农残级，上海安谱实验科技股份有限公司）。

### 1.2 标准溶液的配制

用二氯甲烷将标准品配制成质量浓度为20 mg/L的标准储备液，于‒20°C冰箱中保存；用二氯甲烷稀释标准储备液，配制成质量浓度为1 mg/L的混合标准溶液。临用前再用二氯甲烷逐级稀释混合标准溶液，配制成所需质量浓度。

### 1.3 样品收集

在先前工作^［8，33］^基础上对样品收集流程进行了优化。石英滤膜在使用前置于马弗炉中加热（600 °C）4 h，以去除滤膜上吸附的本底有机物。XAD-2树脂在使用前置于装有二氯甲烷-正己烷（1∶1，v/v）的索氏提取装置中清洗12 h，再使用丙酮进行除水；取0.3 g除水后的XAD-2树脂填入玻璃管（尺寸为8 mm×110 mm）中，用镊子夹取适量的石英棉塞进玻璃管两端，卡紧后放入干燥箱中备用，作为XAD-2树脂采样管。

依次连接石英滤膜、XAD-2树脂采样管与流量可调采样器，将上述装置置于正在焚香的室内环境中，并置于距离香源2 m的范围内，以1 L/min的流速收集颗粒态和气态有机化合物，持续采集30 min。

### 1.4 样品前处理

将收集完样品的石英滤膜裁剪至合适尺寸后装入样品瓶中，加入10 mL二氯甲烷浸泡1 h，随后超声提取20 min，再静置5 min，将提取液转移至50 mL离心管中；重复上述步骤两次，合并提取液；将合并后的提取液氮吹至1 mL，经0.45 μm PTFE滤膜过滤后收集至样品瓶中，待上机分析^［8，33，34］^。取出塞在玻璃管两侧的石英棉，将管中的XAD-2树脂转移至样品瓶中，后续的前处理操作流程与石英滤膜的处理流程一致^［33］^。

### 1.5 非靶向分析条件

#### 1.5.1 色谱条件

色谱柱：HP-5 MS UI超高惰性色谱柱（3 m×0.25 mm， 0.25 µm）；进样口温度为280 ℃；载气为高纯氦气（纯度>99.999%），流量为5 mL/min；不分流进样，进样量为1 μL。柱升温程序：初始温度40 ℃，保持3 min；以5 ℃/min升温至80 ℃，保持2 min；以20 ℃/min升温至180 ℃，保持5 min；以10 ℃/min升温至290 ℃，保持5 min。

#### 1.5.2 质谱条件

离子源：EI源；扫描方式：全扫描（full scan）模式；电离能量：70 eV；灯丝电流：50 μΑ；EI源温度：280 ℃；传输线温度：260 ℃；溶剂延迟：4 min；质量扫描范围：*m/z* 30~400；质量分辨率：60 000 半峰宽（*m/z* 200）。

#### 1.5.3 数据处理

采用Thermo Scientific^TM^ TraceFinder^TM^ 4.1软件（美国Thermo公司）对数据进行采集和处理。在EI全扫描模式下使用GC-Q-Orbitrap-HRMS对样品进行分析。解卷积条件：总离子流（TIC）强度阈值为1×10^7^，质量数偏差容许窗口为±5×10^-6^，信噪比（*S/N*）阈值为3，离子重叠设置为98%，保留时间（retention time， RT）矫正窗口为9 s。在数据处理过程中，先减去空白部分，随后识别每个样品的特征峰，之后在标准质谱库（NIST 2020）中搜索，得到检索得分（SI）、反向检索得分（RSI）、高分辨过滤值（HRF）和反向高分辨过滤值（RHRF）。其中，RSI表示标准谱图与被测谱图的正向匹配指数，RHRF值表示实测质谱中碎片离子的精确质量数及其元素组成与标准谱图库中对应碎片离子的一致性百分比。RHRF和RSI值越高，分析结果的可靠性就越高。基于这两类匹配值，系统会给出综合得分（TS）。用相同的分离方法分析C7~C40正构烷烃，确定各烷烃的RT，并计算未知化合物的保留指数（RI）。通过将未知化合物的RI与标准谱库中化合物的RI进行比较，计算RI偏差（即实测值与质谱库中对应值的差值，ΔRI）。TS越高，ΔRI越低，说明检索结果越可靠。

### 1.6 靶向分析条件

#### 1.6.1 色谱条件

色谱柱：DB-5色谱柱（3 m×0.25 mm， 0.25 µm）；进样口温度为280 ℃；载气为高纯氦气（纯度>99.999%），流量为1 mL/min；不分流进样，进样量为1 μL。柱升温程序：初始温度40 ℃，保持3 min；以5 ℃/min升温至80 ℃，保持2 min；以速度20 ℃/min升温至180 ℃，保持5 min；以10 ℃/min升温至290 ℃，保持10 min。

#### 1.6.2 质谱条件

离子源：EI源；扫描方式：选择离子监测（SIM）模式；离子源温度：230 ℃；接口温度：280 ℃；溶剂延迟：4 min。67种目标化合物的保留时间和质谱参数等信息见表1。

**表1:** 67种目标化合物的保留时间和质谱参数

No.	Compound	CAS No.	RT/min	Quantitative ion （*m/z*）	Qualitative ions （*m/z*）
1	methylbenzene （甲苯）	108-88-3	5.25	91.0^*^	92.0， 65.0
2	isobutyl acetate （乙酸异丁酯）	110-19-0	5.53	43.0^*^	56.0， 73.0
3	ethyl isovalerate （异戊酸乙酯）	108-64-5	7.79	88.0^*^	85.0， 57.0
4	ethylbenzene （乙苯）	100-41-4	7.97	91.0^*^	106.0， 92.0
5	*α*-angelica lactone （*α*-当归内酯）	591-12-8	8.16	55.0^*^	98.0， 43.0
6	paraxylene （对二甲苯）	106-42-3	8.22	91.0^*^	106.0， 105.0
7	*m*-xylene （间二甲苯）	108-38-3	8.22	91.0^*^	106.0， 105.0
8	isopentyl acetate （乙酸异戊酯）	123-92-2	8.52	43.0^*^	70.0， 55.0
9	styrene （苯乙烯）	100-42-5	8.88	104.0^*^	103.0， 78.0
10	1，2-xylene （邻二甲苯）	95-47-6	8.96	91.0^*^	106.0， 105.0
11	cumene （异丙苯）	98-82-8	10.00	105.0^*^	120.0， 79.0
12	*γ*-valerolactone （*γ*-戊内酯）	108-29-2	10.78	56.0^*^	85.0， 41.0
13	phenol （苯酚）	108-95-2	11.90	94.0^*^	66.0， 37.0
14	2-chlorophenol （2-氯苯酚）	95-57-8	12.16	128.0^*^	64.0， 130.0
15	furfuryl acetate （乙酸糠酯）	623-17-6	12.41	81.0^*^	98.0， 52.0
16	*n*-butyl butyrate （丁酸丁酯）	109-21-7	12.51	71.0^*^	89.0， 43.0
17	*cis*-3-hexenyl acetate （乙酸叶醇酯）	3681-71-8	12.96	43.0^*^	67.0， 82.0
18	D-limonene （D-柠檬烯）	5989-27-5	13.73	68.0^*^	93.0， 67.0
19	*γ*-caprolactone （*γ*-己内酯）	108-29-2	14.25	85.0^*^	57.0， 42.0
20	*o*-cresol （2-甲基苯酚）	95-48-7	14.36	108.0^*^	107.0， 79.0
21	isoamyl *n*-butyrate （丁酸异戊酯）	106-27-4	14.42	71.0^*^	70.0， 43.0
22	*p*-cresol （4-甲基苯酚）	106-44-5	14.80	107.0^*^	108.0， 77.0
23	*m*-cresol （3-甲基苯酚）	108-39-4	14.80	107.0^*^	108.0， 77.0
24	allyl hexanoate （己酸烯丙酯）	123-68-2	14.95	99.0^*^	43.0， 71.0
25	methyl benzoate （苯甲酸甲酯）	93-58-3	15.18	105.0^*^	77.0， 136.0
26	isopentyl isopentanoate （异戊酸异戊酯）	659-70-1	15.38	70.0^*^	85.0， 43.0
27	hepty lacetat （乙酸庚酯）	112-06-1	15.49	43.0^*^	70.0， 56.0
28	2，4-dimethylphenol （2.4-二甲基苯酚）	105-67-9	16.01	107.0^*^	122.0， 121.0
29	*γ*-heptalactone （*γ*-庚内酯）	105-21-5	16.05	85.0^*^	56.0， 41.0
30	benzyl acetate （乙酸苄酯）	140-11-4	16.23	108.0^*^	91.0， 43.0
31	2，4-dichlorophenol （2，4-二氯苯酚）	120-83-2	16.29	162.0^*^	164.0， 63.0
32	naphthalene （萘）	91-20-3	16.53	128.0^*^	127.0， 129.0
33	2，6-dichlorophenol （2，6-二氯苯酚）	87-65-0	16.73	162.0^*^	164.0， 63.0
34	ethyl phenylacetate （苯乙酸乙酯）	101-97-3	17.17	91.0^*^	29.0， 164.0
35	phenylethyl acetate （乙酸苯乙酯）	103-45-7	17.30	104.0^*^	43.0， 91.0
36	*γ*-octalactone （*γ*-辛内酯）	104-50-7	17.30	85.0^*^	29.0， 57.0
37	*p*-choro-*m*-cresol （4-氯-3-甲基苯酚）	59-50-7	17.59	107.0^*^	142.0， 77.0
38	ethyl nonanoate （壬酸乙酯）	123-29-5	17.64	88.0^*^	101.0， 43.0
39	menthyl acetate （乙酸薄荷酯）	89-48-5	17.67	95.0^*^	81.0， 43.0
40	2，4，6-trichlorophenol （2，4，6-三氨苯酚）	88-06-2	18.22	196.0^*^	198.0， 97.0
41	2，4，5-trichlorophenol （2，4，5-三氨苯酚）	95-95-4	18.27	196.0^*^	198.0， 97.0
42	geranyl acetate （乙酸香叶酯）	105-87-3	18.38	69.0^*^	41.0， 42.0
43	methyl cinnamate （肉桂酸甲酯）	103-26-4	18.46	131.0^*^	103.0， 162.0
44	anisyl acetate （乙酸茴香酯）	104-21-2	18.73	121.0^*^	120.0， 103.0
45	acenaphthylene （苊烯）	208-96-8	19.21	152.0^*^	151.0， 131.0
46	ethyl cinnamat （肉桂酸乙酯）	103-36-6	19.23	131.0^*^	103.0， 176.0
47	acenaphthene （苊）	83-32-9	19.59	153.0^*^	154.0， 152.0
48	dihydroactinidiolide （二氢猕猴桃内酯）	17092-92-1	20.11	111.0^*^	43.0， 137.0
49	2，3，4，6-tetrachlorophenol （2，3，4，6-四氯酚）	58-90-2	20.24	232.0^*^	230.0， 131.0
50	2，3，4，5-tetrachlorophenol （2，3，4，5-四氯酚）	4901-51-3	20.31	232.0^*^	230.0， 131.0
51	2，3，5，6-tetrachlorophenol （2，3，5，6-四氯酚）	935-95-5	20.35	232.0^*^	230.0， 131.0
52	fluorene （芴）	86-73-7	20.81	166.0^*^	165.0， 167.0
53	methyl dihydrojasmonate （二氢茉莉酮酸甲酯）	24851-98-7	21.59	83.0^*^	82.0， 153.0
54	*γ*-dodecanolactone （*γ*-十二内酯）	2305-05-7	22.05	85.0^*^	55.0， 41.0
55	phenanthrene （菲）	85-01-8	24.31	178.0^*^	176.0， 179.0
56	anthracene （蒽）	120-12-7	24.49	178.0^*^	176.0， 179.0
57	fluoranthene （荧蒽）	206-44-0	28.41	202.0^*^	200.0， 203.0
58	pyren （芘）	129-00-0	29.05	202.0^*^	200.0， 203.0
59	benzo［*a*］anthracene （苯并［*a*］蒽）	56-55-3	32.42	228.0^*^	226.0， 229.0
60	chrysene （䓛）	218-01-9	32.52	228.0^*^	226.0， 229.0
61	benzo［*b*］fluoranthene （苯并［*b*］萤蒽）	205-99-2	35.18	252.0^*^	250.0， 253.0
62	benzo［*k*］fluoranthene （苯并［*k*］萤蒽）	207-08-9	35.18	252.0^*^	250.0， 253.0
63	benzo［*a*］pyrene （苯并［*a*］芘）	50-32-8	35.86	252.0^*^	250.0， 253.0
64	benzo［*e*］pyrene （苯并［*e*］芘）	192-97-2	36.01	252.0^*^	250.0， 253.0
65	indeno［1，2，3-*cd*］pyrene （茚并［1，2，3-*cd*］芘）	193-39-5	40.06	276.0^*^	274.0， 275.0
66	dibenz［*a*，*h*］anthracene （二苯并［*a*，*h*］蒽）	53-70-3	40.21	278.0^*^	276.0， 279.0
67	benzo［*ghi*］perylene （苯并（*ghi*）苝）	191-24-2	41.18	276.0^*^	274.0， 275.0

## 2 结果与讨论

### 2.1 非靶向筛查条件的确定

利用TraceFinder软件（日本岛津公司）的解卷积插件，将基于GC-Q-Orbitrap-HRMS获取的焚香释放物原始数据解析为纯质谱峰。扣除空白背景后，识别每个样品的特征峰，在NIST 2020谱库中进行搜索，针对TIC强度阈值高于1×10⁷的峰开展结构确认和定性分析。质谱匹配指数及RI阈值的选择对于化合物的定性至关重要。若阈值设置过低，会大幅增加后续定性识别的工作量和难度；若阈值设置过高，则可能导致部分化合物丢失。采集的数据经软件处理后，依据分子离子计算其元素组成，并结合二级碎片离子信息预测分子结构，进而得到化合物的质谱匹配指数得分，包括SI、RSI、HRF、RHRF、TS和ΔRI。基于匹配谱库，本研究共检测到70种化合物；通过标准品验证，确认了其中48种化合物（表2）。在空白实际样品中加入含有这48种标准品的混合标准溶液（500 µg/L），开展阈值测试工作。理论上，当SI≥600、RSI≥600、HRF≥80.0、RHRF≥80.0、TS≥90.0及ΔRI≤40时，鉴定结果的准确性较高^［33，34］^。如表2所示，48种化合物的阈值测试结果均在理论范围内，其中甲苯色谱峰的保留时间早于C7~C40正构烷烃标样范围，导致无法计算其ΔRI值。通过合理设置阈值，能够有效降低假阳性结果出现的概率，提高非靶向筛查的效率。因此，本实验将非靶向筛查条件设定为SI≥600、RSI≥600、HRF≥80.0、RHRF≥80.0、TS≥90.0及ΔRI≤40。

**表2:** 48种化合物的RT、SI、RSI、HRF、RHRF、TS和ΔRI

Compound	RT/min	SI	RSI	HRF	RHRF	TS	ΔRI
Methylbenzene	4.69	925	929	99.5	100.0	98.3	/
Ethylbenzene	7.23	912	914	99.8	100.0	98.2	12
*p*-Xylene	7.47	906	907	99.9	100.0	98.1	11
Styrene	8.13	951	953	99.7	100.0	98.9	10
*m*-Xylene	8.19	882	883	100.0	100.0	97.7	20
Cumene	9.21	783	783	99.5	99.5	95.5	0
*o*-Cresol	13.72	901	912	99.0	99.2	97.6	14
*p*-Cresol	14.25	795	803	98.4	98.5	95.3	5
Isopentyl isopentanoate	14.90	752	752	99.8	100.0	95.0	3
Hepty lacetat	15.03	715	785	96.6	99.6	93.0	4
2，4-Dimethylphenol	15.59	917	918	99.4	99.6	98.1	7
*γ*-Heptalactone	15.69	678	679	99.7	99.9	93.5	4
Benzyl acetate	15.84	810	815	99.5	100.0	96.0	6
2，4-Dichlorophenol	15.87	920	922	95.6	96.0	96.7	3
Naphthalene	16.11	913	914	93.0	95.2	95.5	1
2，6-Dichlorophenol	16.33	875	884	99.0	98.7	97.1	14
Ethyl phenylacetate	16.81	833	837	99.6	100.0	96.5	3
Phenylethyl acetate	16.94	890	890	99.9	99.9	97.8	5
*γ*-Octalactone	16.96	769	769	99.7	99.7	95.3	4
*p*-Choro-*m*-cresol	17.25	924	927	99.3	99.7	98.2	4
Ethyl nonanoate	17.29	698	698	99.8	99.8	93.9	1
Menthyl acetate	17.31	625	626	99.3	99.4	92.2	3
2，4，6-Trichlorophenol	17.86	905	907	98.2	98.8	97.4	9
2，4，5-Trichlorophenol	17.91	869	870	96.6	96.5	96.0	11
Geranyl acetate	18.03	785	786	100.0	100.0	95.7	2
Methyl cinnamate	18.10	832	837	99.5	99.6	96.5	12
Anisyl acetate	18.36	852	853	99.9	99.9	97.0	1
Acenaphthylene	18.75	904	939	91.3	98.2	94.6	14
Ethyl cinnamate	18.79	805	840	99.5	99.9	95.9	10
Acenaphthene	19.09	924	946	92.5	95.6	95.5	15
Dihydroactinidiolide	19.56	844	846	99.7	99.8	96.8	20
2，3，4，6-Tetrachlorophenol	19.67	892	903	92.7	96.9	94.9	4
2，3，5，6-Tetrachlorophenol	19.76	894	902	91.2	97.9	94.4	16
Fluorene	20.15	881	886	82.7	86.8	90.7	14
Methyl dihydrojasmonate	20.87	737	738	99.8	99.9	94.7	3
Phenanthrene	23.35	912	924	96.6	97.6	96.9	24
Anthracene	23.54	903	910	96.9	97.7	96.9	24
Fluoranthene	27.66	924	932	90.3	94.0	94.6	3
Pyrene	28.32	913	935	90.1	94.6	94.3	28
Benz［*a*］anthracene	31.73	869	883	88.9	92.6	93.0	1
Chrysene	31.83	891	901	88.6	92.7	93.3	5
Benzo［*b*］fluoranthene	34.33	893	901	92.9	96.2	95.0	9
Benzo［*k*］fluoranthene	34.38	845	852	91.0	91.1	93.3	33
Benzo［*a*］pyrene	34.90	876	923	90.4	91.9	93.7	12
Benzo［*e*］pyrene	35.01	848	859	88.9	91.6	92.5	35
Indeno［1，2，3-*cd*］pyrene	37.73	866	888	90.6	92.8	93.6	27
Dibenz［*a*，*h*］anthracene	37.83	829	830	91.2	92.9	93.1	29
Benzo［*ghi*］perylene	38.44	863	873	88.6	91.7	92.7	36

The data in this table were obtained through non-targeted analysis. /： no value.

### 2.2 分析条件的优化

#### 2.2.1 质谱条件的优化

首先在GC-MS的全扫描模式下，设定扫描*m*/*z*范围为25~300，对含有67种目标化合物的混合标准溶液（0.5 mg/L）进行分析检测。通过将TIC色谱图与已知标准物质的特征图谱进行对比，确认不同保留时间的色谱峰对应的目标化合物。选取仪器响应值最高的离子作为定量离子，响应值次高的两个离子作为定性离子，用于辅助定性分析。在将全扫描模式下检测到的化合物全部识别后，利用已确定的定性离子和定量离子构建靶向分析方法。实验采用同一根DB-5色谱柱（3 m×0.25 mm， 0.25 µm），在相同的色谱与质谱条件下，分别使用气相色谱-三重四极杆质谱联用仪和气相色谱-质谱联用仪进行测定。以67种目标化合物（500 µg/L）的*S/N*为考察指标，结果表明，采用气相色谱-质谱联用仪所测得的*S/N*明显高于气相色谱-三重四极杆质谱联用仪（相关数据见附表1，www.chrom-China.com），且各目标化合物在气相色谱-质谱联用仪上的分离效果更佳、峰形更好。因此，最终选择利用GC-MS来建立目标化合物的靶向分析方法。

#### 2.2.2 色谱条件的优化

以GC-MS作为检测仪器，实验比较了DB-5柱（3 m×0.25 mm， 0.25 µm）和SH-5Sil MS柱（3 m×0.25 mm， 0.25 µm）两种色谱柱对67种目标化合物响应值和分离效果的影响。结果表明，大部分目标化合物在DB-5色谱柱上的分离效果更佳、峰形更好且响应值更高；同时，DB-5色谱柱能够更快速地检测出所有化合物，因此选择该色谱柱用于后续实验。67种目标化合物经DB-5色谱柱分离后的TIC色谱图见图1。其中，对二甲苯与间二甲苯、3-甲基苯酚与4-甲基苯酚、苯并［*b*］荧蒽与苯并［*k*］荧蒽这3组同分异构体无法完全分离，故将它们作为一个整体进行积分处理。

**图1:**
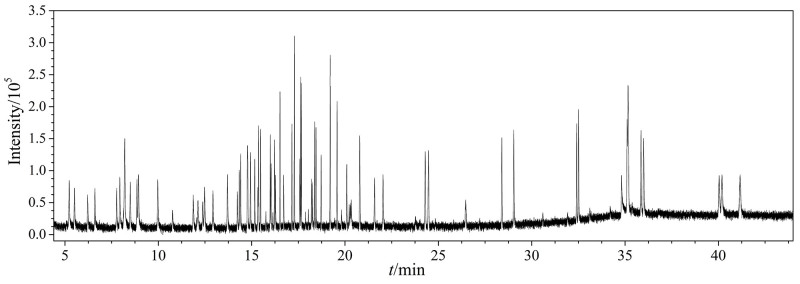
67种目标化合物经DB-5色谱柱分离后的TIC色谱图

### 2.3 方法学验证

#### 2.3.1 线性范围、检出限及定量限

配制系列质量浓度（10、20、50、100、200、500 µg/L）的67种有机化合物混合标准溶液，按1.6节条件进样分析。以各目标化合物的质量浓度为横坐标（*x*， µg/L），定量离子峰面积为纵坐标（*y*），绘制标准曲线，得到线性回归方程。结果如表3所示，67种有机化合物在10~500 µg/L范围内线性关系良好，相关系数（*r*）均≥0.999 0。分别以3倍和10倍*S/N*确定检出限（LOD）和定量限（LOQ），67种有机化合物的LOD和LOQ分别为0.02~0.33 µg/m^3^和0.03~0.67 µg/m^3^。

**表3:** 67种有机化合物的回归方程、线性范围、相关系数、检出限和定量限

No.	Compound	Regression equation	Linear range/（μg/L）	*r*	LOD/（µg/m^3^）	LOQ/（µg/m^3^）
1	methylbenzene	*Y*=297.75*X*+902.46	10‒500	0.9991	0.03	0.07
2	sobutyl acetate	*Y*=174.65*X*‒402.83	10‒500	0.9993	0.07	0.17
3	ethyl isovalerate	*Y*=87.14*X‒*8.61	10‒500	0.9991	0.07	0.17
4	ethylbenzene	*Y*=328.91*X*+590.33	10‒500	0.9992	0.03	0.07
5	*α*-angelica lactone	*Y*=51.37*X‒*251.76	10‒500	0.9992	0.17	0.33
6	paraxylene	*Y*=548.81*X*+1936.15	10‒500	0.9992	0.02	0.03
7	*m*-xylene	*Y*=548.81*X*+1936.15	10‒500	0.9992	0.02	0.03
8	isopentyl acetate	*Y*=127.98*X‒*81.70	10‒500	0.9991	0.07	0.17
9	styrene	*Y*=205.39*X*+907.85	10‒500	0.9996	0.02	0.03
10	1，2-xylene	*Y*=276.56*X*+511.88	10‒500	0.9995	0.07	0.17
11	cumene	*Y*=278.01*X‒*322.05	10‒500	0.9996	0.03	0.07
12	*γ*-valerolactone	*Y*=70.86*X‒*397.15	10‒500	0.9997	0.17	0.33
13	phenol	*Y*=181.82*X‒*349.79	10‒500	0.9994	0.07	0.17
14	2-chlorophenol	*Y*=141.18*X‒*567.11	10‒500	0.9992	0.07	0.17
15	furfuryl acetate	*Y*=69.38*X*+36.34	10‒500	0.9993	0.17	0.33
16	*n*-butyl butyrate	*Y*=107.48*X*+15.26	10‒500	0.9991	0.07	0.17
17	*cis*-3-bexenyl acetate	*Y*=116.20*X‒*31.27	10‒500	0.9992	0.07	0.17
18	D-limonene	*Y*=77.93*X*+58.60	10‒500	0.9995	0.07	0.17
19	*γ*-caprolactone	*Y*=150.28*X*+8.29	10‒500	0.9994	0.03	0.07
20	*o*-cresol	*Y*=123.46*X‒*380.06	10‒500	0.9994	0.07	0.17
21	bsoamyl *n*-butyrate	*Y*=107.57*X‒*67.38	10‒500	0.9997	0.07	0.17
22	*p*-cresol	*Y*=258.35*X‒*1055.72	10‒500	0.9995	0.03	0.07
23	*m*-cresol	*Y*=258.35*X‒*1055.72	10‒500	0.9995	0.03	0.07
24	allyl hexanoate	*Y*=75.56*X‒*70.42	10‒500	0.9993	0.07	0.17
25	methyl benzoate	*Y*=176.97*X*+179.85	10‒500	0.9990	0.03	0.07
26	isopentyl isopentanoate	*Y*=260.84*X‒*488.11	10‒500	0.9993	0.03	0.07
27	hepty lacetat	*Y*=104.87*X*+15.79	10‒500	0.9993	0.07	0.17
28	2，4-dimethylphenol	*Y*=131.16*X‒*530.18	10‒500	0.9995	0.03	0.07
29	*γ*-heptalactone	*Y*=216.56*X‒*155.49	10‒500	0.9992	0.03	0.07
30	benzyl acetate	*Y*=111.68*X‒*50.84	10‒500	0.9993	0.03	0.07
31	2，4-dichlorophenol	*Y*=87.58*X‒*722.39	10‒500	0.9995	0.07	0.17
32	naphthalene	*Y*=418.05*X*+199.10	10‒500	0.9990	0.02	0.03
33	2，6-dichlorophenol	*Y*=86.70*X‒*645.59	10‒500	0.9996	0.07	0.17
34	ethyl phenylacetate	*Y*=297.51*X‒*180.06	10‒500	0.9992	0.02	0.03
35	phenylethyl acetate	*Y*=210.50*X‒*216.75	10‒500	0.9993	0.02	0.03
36	*γ*-octalactone	*Y*=254.00*X‒*473.84	10‒500	0.9993	0.03	0.07
37	*p*-choro-*m*-cresol	*Y*=104.97*X‒*740.20	10‒500	0.9996	0.07	0.17
38	cthyl nonanoate	*Y*=152.55*X‒*577.97	10‒500	0.9993	0.03	0.07
39	menthyl acetate	*Y*=118.95*X‒*206.85	10‒500	0.9995	0.07	0.17
40	2，4，6-trichlorophenol	*Y*=53.95*X‒*507.71	10‒500	0.9996	0.07	0.17
41	2，4，5-trichlorophenol	*Y*=67.33*X‒*794.94	10‒500	0.9994	0.07	0.17
42	geranyl acetate	*Y*=97.90*X‒*792.13	10‒500	0.9996	0.03	0.07
43	methyl cinnamate	*Y*=131.67*X‒*371.53	10‒500	0.9994	0.03	0.07
44	anisyl acetate	*Y*=108.54*X‒*1032.51	10‒500	0.9998	0.03	0.07
45	acenaphthylene	*Y*=348.57*X‒*1011.07	10‒500	0.9995	0.03	0.07
46	ethyl cinnamate	*Y*=155.74*X‒*578.78	10‒500	0.9995	0.03	0.07
47	acenaphthene	*Y*=221.97*X‒*306.09	10‒500	0.9993	0.03	0.07
48	dihydroactinidiolide	*Y*=87.02*X‒*204.72	10‒500	0.9995	0.03	0.07
49	2，3，4，6-tetrachlorophenol	*Y*=33.43*X‒*367.11	10‒500	0.9996	0.33	0.67
50	2，3，4，5-tetrachlorophenol	*Y*=37.83*X‒*543.84	10‒500	0.9992	0.33	0.67
51	2，3，5，6-tetrachlorophenol	*Y*=41.45*X‒*394.10	10‒500	0.9996	0.33	0.67
52	fluorene	*Y*=258.74*X‒*602.55	10‒500	0.9995	0.03	0.07
53	methyl dihydrojasmonate	*Y*=126.53*X‒*764.41	10‒500	0.9997	0.03	0.07
54	*γ*-dodecanolactone	*Y*=183.97*X‒*1350.18	10‒500	0.9997	0.07	0.17
55	phenanthrene	*Y*=383.27*X‒*1226.96	10‒500	0.9996	0.03	0.07
56	anthracene	*Y*=390.03*X‒*2013.38	10‒500	0.9997	0.03	0.07
57	fluoranthene	*Y*=405.33*X‒*2017.02	10‒500	0.9997	0.03	0.07
58	pyrene	*Y*=434.48*X‒*2419.71	10‒500	0.9997	0.03	0.07
59	benzo［*a*］anthracene	*Y*=410.11*X‒*2596.59	10‒500	0.9997	0.03	0.07
60	chrysene	*Y*=438.45*X‒*2456.70	10‒500	0.9997	0.03	0.07
61	benzo［*b*］fluoranthene	*Y*=969.61*X‒*7186.94	10‒500	0.9997	0.03	0.07
62	benzo［*k*］fluoranthene	*Y*=969.61*X‒*7186.94	10‒500	0.9997	0.03	0.07
63	benzo［*a*］pyrene	*Y*=472.43*X‒*3700.61	10‒500	0.9997	0.03	0.07
64	benzo［*e*］pyrene	*Y*=490.36*X‒*3964.96	10‒500	0.9998	0.03	0.07
65	indeno［1，2，3-*cd*］pyrene	*Y*=428.48*X‒*4315.63	10‒500	0.9996	0.07	0.17
66	dibenz［*a*，*h*］anthracene	*Y*=498.39*X‒*5753.07	10‒500	0.9995	0.07	0.17
67	benzo［*ghi*］perylene	*Y*=499.41*X‒*5052.12	10‒500	0.9997	0.07	0.17

*Y*： peak area； *X*： mass concentration， μg/L.

#### 2.3 2　回收率和精密度

向全新的石英滤膜和XAD-2树脂中分别添加低、中、高3个水平（50、100、200 μg/L）的67种有机化合物混合标准溶液，进行加标回收试验，每个加标水平平行测定6次，计算回收率和相对标准偏差（RSD）。实验结果表明，在3个加标水平下，除四氯苯酚的3种同分异构体在XAD-2树脂中无法检出（可能是由于二氯甲烷的极性不足以有效破坏四氯苯酚及其同分异构体与XAD-2树脂间的疏水作用），其余64种有机化合物在XAD-2树脂中的回收率为72.7%~119.0%，RSD为0.9%~4.1%；67种有机化合物在石英滤膜中的回收率为71.5%~118.9%，RSD为0.7%~9.5%（相关数据见附表2）。实验结果表明，该方法的准确度和精密度符合方法学验证要求。

### 2.4 方法比对

将本方法与现有方法进行比较，结果如表4所示。本方法步骤简单，可检测的化合物数量较多，同时具有较好的回收率和较低的LOD；本方法采用外标法定量，相较于内标法，降低了实验成本；本方法利用自制的XAD-2树脂采样管收集焚香气态组分，在准确度和精密度均符合方法学验证要求的情况下，能够大幅降低实验室焚香气体收集装置的成本。

**表4:** 本方法与其他方法的比较

Ref.	Numbers of analytes	Matrix	Pretreatment method	Quantitative analysis method	Detection method	Recovery/%	LOD/（µg/m^3^）
［^35^］	16	incense	ultrasonic extraction	internal standard method	GC-MS	76.6‒118.9	0.01‒0.06
［^36^］	67	ambient air	thermal desorption	internal standard method	GC-MS	81.6‒114.9	0.3‒2.4
［^37^］	22	ambient air	thermal desorption	external standard method	GC-MS	90.3‒112.0	2‒5
This method	67	incense	ultrasonic extraction	external standard method	GC-MS	71.5‒119.0	0.02‒0.33

### 2.5 实际样品测定

利用本文所建方法对室内焚香样品（Joss 1、Joss 2、Powder 1、Powder 2）释放的颗粒物及气体中的有机化合物含量和组成进行分析，其中Joss为两类燃烧线香，Powder为两类电加热（200 ℃）香粉。结果显示，在相同的焚香时间（30 min）内，线香点燃时释放的气态和颗粒态有机化合物总含量均高于电加热香粉（图2），这可能是由于电加热的温度相对较低，不足以引发剧烈的热解或氧化反应。由表5和图2可知，线香燃烧产生的颗粒态有机化合物以酚类化合物（Joss 1： 497.8 µg/m^3^，Joss 2： 97.5 µg/m^3^）和多环芳烃（Joss 1： 211.5 µg/m^3^，Joss 2： 95.8 µg/m^3^）为主，其次是酯类化合物（Joss 1： 44.1 µg/m^3^，Joss 2： 7.5µg/m^3^），苯系物（Joss 1： 1.8 µg/m^3^，Joss 2： 0.7 µg/m^3^）仅占很少一部分；其中，多环芳烃从二环到六环均有释放，且以三环化合物占主导地位（以菲的含量（Joss 1： 94.8 µg/m^3^，Joss 2： 37.9 µg/m^3^）最高）。电加热香粉产生的颗粒态有机化合物组成与线香相似，以酚类化合物（Powder 1： 38.5 µg/m^3^，Powder 2： 9.7 µg/m^3^）和多环芳烃（Powder 1： 13.9 µg/m^3^，Powder 2： 27.4 µg/m^3^）为主，其次是酯类化合物（Powder 1： 3.1 µg/m^3^，Powder 2： 11.5 µg/m^3^）和苯系物（Powder 1： 0.4 µg/m^3^，Powder 2： 0.8 µg/m^3^）。在焚烧线香释放的气态有机化合物中检测到了大量的苯系物（Joss 1： 6753.1 µg/m^3^，Joss 2： 3662.9 µg/m^3^），其次是酚类化合物（Joss 1： 479.2 µg/m^3^，Joss 2： 699.0 µg/m^3^）、酯类化合物（Joss 1： 208.5 µg/m^3^，Joss 2： 174.1 µg/m^3^）和多环芳烃（Joss 1： 117.5 µg/m^3^，Joss 2： 131.3 µg/m^3^）。电加热香粉释放的气态有机化合物以酚类化合物（Powder 1： 435.1 µg/m^3^，Powder 2： 247.5 µg/m^3^）和苯系物（Powder 1： 360.8 µg/m^3^，Powder 2： 88.4 µg/m^3^）为主，其次是酯类化合物（Powder 1： 139.6 µg/m^3^，Powder 2： 101.0 µg/m^3^）和多环芳烃（Powder 1： 67.1 µg/m^3^，Powder 2： 44.0 µg/m^3^）。

**表5:** 室内焚香样品（两类线香和两类电加热香粉）释放的颗粒态及气态有机化合物的含量测定结果

Category	Compound	Joss 1		Joss 2		Powder 1		Powder 2	
		Particulate	Gaseous	Particulate	Gaseous	Particulate	Gaseous	Particulate	Gaseous
Benzene series	methylbenzene	0.6	3242.5	0.5	1434.0	0.4	74.4	0.4	16.0
	ethylbenzene	ND	432.9	ND	291.1	ND	32.5	0.1	ND
	paraxylene	0.2	654.9	ND	401.7	ND	46.8	ND	8.8
	*m*-xylene	0.2	654.9	ND	401.7	ND	46.8	ND	8.8
	styrene	0.6	1310.1	0.2	831.1	ND	118.8	0.3	46.5
	1，2-xylene	0.2	400.8	ND	279.7	ND	34.5	ND	5.8
	cumene	ND	57.0	ND	23.6	ND	7.0	ND	2.5
Esters	isobutyl acetate	ND	ND	ND	ND	ND	ND	ND	7.0
	ethyl isovalerate	ND	ND	ND	2.1	ND	ND	ND	ND
	*α*-angelica lactone	ND	ND	ND	ND	ND	ND	ND	ND
	isopentyl acetate	ND	ND	ND	ND	ND	ND	ND	ND
	*γ*-valerolactone	ND	ND	ND	ND	ND	15.4	ND	ND
	furfuryl acetate	ND	ND	ND	ND	ND	ND	ND	ND
	*n*-butyl butyrate	ND	ND	ND	ND	ND	ND	ND	ND
	*cis*-3-hexenyl acetate	ND	ND	ND	ND	ND	ND	ND	ND
	D-limonene	ND	149.7	ND	140.4	0.8	103.1	ND	70.2
	*γ*-caprolactone	ND	ND	ND	ND	ND	ND	ND	ND
	isoamyl *n*-butyrate	ND	ND	ND	ND	ND	ND	ND	ND
	allyl hexanoate	ND	ND	ND	ND	ND	ND	ND	ND
	methyl benzoate	ND	8.0	ND	8.4	ND	5.9	ND	3.2
	isopentyl isopentanoate	2.9	ND	3.3	ND	1.0	ND	0.2	2.5
	hepty lacetat	ND	ND	ND	ND	ND	ND	ND	ND
	*γ*-heptalactone	ND	ND	ND	ND	ND	ND	ND	ND
	benzyl acetate	10.8	ND	ND	ND	ND	ND	ND	ND
	ethyl phenylacetate	ND	3.6	ND	4.5	ND	1.8	ND	1.4
	phenylethyl acetate	ND	ND	ND	ND	ND	ND	ND	ND
	*γ*-octalactone	1.0	ND	0.3	ND	ND	ND	ND	0.9
	ethyl nonanoate	ND	ND	ND	ND	ND	ND	ND	ND
	menthyl acetate	ND	ND	ND	ND	ND	6.6	ND	9.2
	geranyl acetate	8.7	24.2	ND	18.7	ND	6.8	11.3	6.6
	methyl cinnamate	20.7	7.3	ND	ND	1.3	ND	ND	ND
	anisyl acetate	ND	ND	ND	ND	ND	ND	ND	ND
	ethyl cinnamate	ND	ND	3.9	ND	ND	ND	ND	ND
	dihydroactinidiolide	ND	15.7	ND	ND	ND	ND	ND	ND
	methyl dihydrojasmonate	ND	ND	ND	ND	ND	ND	ND	ND
	*γ*-dodecanolactone	ND	ND	ND	ND	ND	ND	ND	ND
Phenols	phenol	248.9	343.3	32.7	498.8	15.9	327.5	3.6	167.4
	2-chlorophenol	ND	0.4	ND	6.5	ND	3.1	ND	0.8
	*o*-cresol	39.6	39.1	8.8	54.9	3.3	32.3	0.7	20.2
	*p*-cresol	84.6	39.7	22.7	60.3	7.9	36.1	2.0	24.6
	*m*-cresol	84.6	39.7	22.7	60.3	7.9	36.1	2.0	24.6
	2，4-dimethylphenol	36.1	17.0	10.0	18.2	3.2	ND	1.1	9.9
	2，4-dichlorophenol	ND	ND	ND	ND	ND	ND	ND	ND
	2，6-dichlorophenol	ND	ND	0.6	ND	0.3	ND	0.3	ND
	*p*-choro-*m*-cresol	4.0	ND	ND	ND	ND	ND	ND	ND
	2，4，6-trichlorophenol	ND	ND	ND	ND	ND	ND	ND	ND
	2，4，5-trichlorophenol	ND	ND	ND	ND	ND	ND	ND	ND
	2，3，4，6-tetrachlorophenol	ND	ND	ND	ND	ND	ND	ND	ND
	2，3，4，5-tetrachlorophenol	ND	ND	ND	ND	ND	ND	ND	ND
	2，3，5，6-tetrachlorophenol	ND	ND	ND	ND	ND	ND	ND	ND
PAHs	naphthalene	11.2	113.5	1.5	126.0	0.4	66.1	0.2	42.7
	acenaphthylene	18.7	ND	ND	ND	ND	ND	ND	ND
	acenaphthene	3.8	1.5	ND	1.1	ND	ND	ND	0.3
	fluorene	ND	ND	ND	1.1	ND	ND	ND	ND
	phenanthrene	94.8	2.3	37.9	2.8	8.7	0.7	ND	0.4
	anthracene	ND	ND	ND	ND	ND	0.3	ND	0.2
	fluoranthene	ND	ND	15.0	0.3	2.6	ND	23.9	0.2
	pyrene	22.6	0.2	13.6	ND	1.2	ND	0.9	ND
	benzo［*a*］anthracene	14.2	ND	6.4	ND	0.3	ND	0.6	ND
	chrysene	22.8	ND	13.0	ND	0.2	ND	1.1	0.2
	benzo［*b*］fluoranthene	2.6	ND	1.2	ND	ND	ND	ND	ND
	benzo［*k*］fluoranthene	2.6	ND	1.2	ND	ND	ND	ND	ND
	benzo［*a*］pyrene	4.0	ND	ND	ND	0.5	ND	0.7	ND
	benzo［*e*］pyrene	6.0	ND	3.2	ND	ND	ND	ND	ND
	indeno［1，2，3-*cd*］pyrene	4.4	ND	2.1	ND	ND	ND	ND	ND
	fibenz［*a*，*h*］anthracene	1.1	ND	0.7	ND	ND	ND	ND	ND
	benzo［*ghi*］perylene	2.7	ND	ND	ND	ND	ND	ND	ND

ND： not detected.

**图2:**
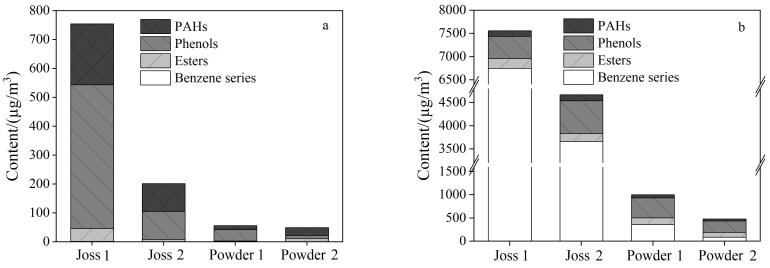
4种焚香样品释放的（a）颗粒态有机化合物和（b）气态有机化合物组成含量图

由表5可知，在线香燃烧释放的气态有机化合物中，甲苯（Joss 1： 3242.5 µg/m^3^，Joss 2： 1434.0 µg/m^3^）是苯系物中含量最高的化合物，其次是苯乙烯（Joss 1： 1310.1 µg/m^3^，Joss 2： 831.1 µg/m^3^）；而对于电加热香粉，苯乙烯（Powder 1： 118.8 µg/m^3^，Powder 2： 46.5 µg/m^3^）是苯系物中含量最高的化合物。在4种焚香样品释放的气态有机化合物中，苯酚（Joss 1： 343.3 µg/m^3^，Joss 2： 498.8 µg/m^3^，Powder 1： 327.5 µg/m^3^，Powder 2： 167.4 µg/m^3^）是酚类化合物中含量最高的化合物。在4种焚香样品释放的气态有机化合物中，酯类化合物D-柠檬烯均有大量检出（Joss 1： 149.7 µg/m^3^，Joss 2： 140.4 µg/m^3^，Powder 1： 103.1 µg/m^3^，Powder 2： 70.2 µg/m^3^）。此外，在电加热香粉释放的气态组分中检出了乙酸异丁酯（Joss 2： 7.0 µg/m³），在线香样品释放的气态组分中检出了肉桂酸甲酯（Joss 1： 7.3 µg/m³）。

上述结果表明，不同种类的香在不同焚烧方式下释放的有机化合物种类不同，含量也存在较大差异。

## 3 结论

本研究建立了焚香释放气体及颗粒物中有机化合物的非靶向分析方法，并在此基础上利用超声提取-GC-MS技术，建立了可同时测定焚香释放的67种有机化合物的靶向分析方法。该方法简单、快速，且对仪器设备的要求较低。但是，目前本研究仅检测了焚香产生的部分代表性产物，在未来工作中还需丰富和完善该方法所涵盖的有机化合物类别与数量，从而为焚香造成的室内空气污染监测与风险评估提供更有力的技术支持。

## References

[1] Wang X， Chan A W H . Environ Sci Technol， 2023， 57（45）： 17384 37927234 10.1021/acs.est.3c04639

[2] Davies H L， O′Leary C， Dillon T， et al . Environ Sci Process Impacts， 2023， 25（9）： 1532 37609942 10.1039/d3em00167a

[3] Chuang H C， Jones T， Chen Y， et al . Anal Bioanal Chem， 2011， 401（10）： 3095 21769554 10.1007/s00216-011-5209-7

[4] Ho S S， Yu J Z . J Environ Monit， 2002， 4（5）： 728 12400922 10.1039/b200998f

[5] Yang T T， Lin T S， Chang M B . Environ Contam Tox， 2007， 78（5）： 308 10.1007/s00128-007-9184-9 17618388

[6] Shrestha O . J Nepal Med Assoc， 2020， 58（230）： 823 10.31729/jnma.5286 PMC7654492 34504363

[7] Manoukian A， Buiron D， Temime-Roussel B， et al . Environ Sci Pollut Res Int， 2016， 23（7）： 6300 26614451 10.1007/s11356-015-5819-2

[8] Li S H， Hao Z R， Shen H L， et al . Atmos Pollut Res， 2023， 14（8）： 101811

[9] Song K， Tang R， Li A， et al . Sci Total Environ， 2023， 897： 165319 37414164 10.1016/j.scitotenv.2023.165319

[10] Lee S C， Wang B . Atmos Environ， 2004， 38（7）： 941

[11] Yang T T， Lin S T， Shie R H， et al . Aerosol Air Qual Res， 2016， 16（10）： 2570

[12] Yang T T， Lin S T， Lin T S， et al . Sci Total Environ， 2015， 506/507： 391 25460974 10.1016/j.scitotenv.2014.11.029

[13] Song K， Tang R Z， Zhang J S， et al . Atmos Chem Phys， 2023， 23（21）： 13585

[14] Lu F， Li S， Shen B， et al . J Hazard Mater， 2020， 384： 121428 31699486 10.1016/j.jhazmat.2019.121428

[15] Liu C G， Chen D D， Chen X E . Environ Sci Technol， 2022， 56（5）： 2897 35188384 10.1021/acs.est.1c06535

[16] Zhang J P， Chen W J， Li J N， et al . Procedia Eng， 2015， 121： 992 10.1016/j.proeng.2015.09.023 PMC7128962 32288921

[17] Yadav V K， Malik P， Tirth V， et al . J Inflamm Res， 2022， 15： 2665 35509323 10.2147/JIR.S347489 PMC9058426

[18] Wong A， Lou W， Ho K F， et al . Sci Rep， 2020， 10（1）： 7090 32341386 10.1038/s41598-020-63568-6 PMC7184605

[19] He Y L， Fan W M， Liu T J， et al . Chinese Journal of Health Laboratory Technology， 2022， 32（14）： 1699

[20] Cai M Z . Chinese Journal of Chromatography， 2022， 40（12）： 1111 36450351 10.3724/SP.J.1123.2022.01022 PMC9727747

[21] Mao W W . Bao-Steel Technology， 2024（3）： 42

[22] Guan X L， Duan X Y . Chemical Engineering & Equipment， 2024（1）： 126

[23] Kao W Y， Hsiang C Y， Ho S C， et al . Molecules， 2018， 23（11）： 2969 30441810 10.3390/molecules23112969 PMC6278519

[24] HJ 638-2012

[25] GB 41700-2022

[26] HJ 583-2010

[27] Zheng Q Y， Zhi Y J， Duan W J， et al . Chinese Journal of Chromatography， 2024， 42（9）： 909 39198950 10.3724/SP.J.1123.2023.12015 PMC11358869

[28] Kwiecien N W， Bailey D J， Rush M J， et al . Anal Chem， 2015， 87（16）： 8328 26192401 10.1021/acs.analchem.5b01503 PMC5176256

[29] Misra B B， Olivier M . J Proteome Res， 2020， 19（7）： 2717 31978300 10.1021/acs.jproteome.9b00774 PMC9701538

[30] Mol H G J， Tienstra M， Zomer P . Anal Chim Acta， 2016， 935： 161 27543025 10.1016/j.aca.2016.06.017

[31] Sapozhnikova Y . Talanta， 2021， 226： 122120 33676675 10.1016/j.talanta.2021.122120

[32] Yang L， Wang S， Peng X， et al . Sci Total Environ， 2019， 664： 107 30739845 10.1016/j.scitotenv.2019.02.001

[33] Liu Y H， Zhu G H， Yu Z F， et al . Environ Sci Technol， 2024， 58（41）： 18356 39264101 10.1021/acs.est.4c06169

[34] Liu Y H， Li H Y， Kong X W， et al . Journal of Chinese Mass Spectrometry Society， 2023， 44（1）： 66

[35] Yang T T， Lin S T， Hung H F， et al . Aerosol Air Qual Res， 2013， 13（2）： 662

[36] Zhu X P， Ma H L， Zhu X H， et al . Chinese Journal of Chromatography， 2019， 37（11）： 1228 31642277 10.3724/SP.J.1123.2019.04043

[37] Feng S L， Wan Z H， Xia J D， et al . Sichuan Environment， 2021， 40（3）： 64

